# Acute Limb Ischemia During Pregnancy: A Case Report and Narrative Review of the Literature

**DOI:** 10.3390/jcm15187188

**Published:** 2026-09-16

**Authors:** Lorenzo Annesi, Erna Saric, Giovanni Tossetta, Christina Stern, Davide Agnoletti, Luciano Potena, Claudio Borghi, Federica Piani

**Affiliations:** 1 Department of Medical and Surgical Sciences, University of Bologna, 40138 Bologna, Italy; davide.agnoletti@unibo.it (D.A.); luciano.potena2@unibo.it (L.P.); claudio.borghi@unibo.it (C.B.); 2 Cardiovascular Internal Medicine Unit, IRCCS Azienda Ospedaliero-Universitaria di Bologna, 40138 Bologna, Italy; 3 Division of Physiology and Pathophysiology, Otto Loewi Research Center for Vascular Biology, Immunology and Inflammation, Medical University of Graz, 8010 Graz, Austria; erna.saric@medunigraz.at (E.S.); federica.piani@medunigraz.at (F.P.); 4 Department for the Promotion of Human Science and Quality of Life, San Raffaele Roma University, 00166 Rome, Italy; giovanni.tossetta@uniroma5.it; 5 Department of Obstetrics and Gynaecology, University Hospital Graz, 8036 Graz, Austria; christina.stern@medunigraz.at

**Keywords:** arterial thrombosis, acute limb ischemia, pregnancy, preeclampsia, hypertensive disorders of pregnancy

## Abstract

**Background**: Pregnancy is a well-recognized hypercoagulable state that increases the risk of thrombotic complications, most commonly venous thromboembolism. In contrast, arterial thrombosis, including acute limb ischemia (ALI), is exceedingly rare but potentially catastrophic, and no specific recommendations address its management during pregnancy. **Methods**: We report the clinical presentation of a pregnant woman who developed ALI during early gestation, along with a narrative review of the available literature. **Results**: Available evidence on ALI during pregnancy is limited to isolated case reports, predominantly occurring in late gestation or postpartum, with only two cases described in the first or second trimester, both related to popliteal artery entrapment. Cardioembolic events secondary to peripartum cardiomyopathy, often associated with preeclampsia, represent the most frequently reported etiology, whereas in situ arterial thrombosis is uncommon and typically occurs in the presence of thrombophilia or autoimmune disease. We report the first documented case of ALI in early pregnancy occurring in the absence of structural vascular abnormalities, thrombophilic disorders or identifiable cardioembolic triggers, despite the presence of multiple clinical cardiovascular risk factors. The clinical course was further complicated by preeclampsia and postpartum hemorrhage. **Conclusions**: This case suggests that pregnancy-related prothrombotic changes may contribute to acute arterial thrombosis even in early gestation in the presence of an increased burden of clinical risk factors. We highlight the importance of comprehensive thrombotic risk stratification and the development of evidence-based management strategies for pregnancy-associated arterial thrombosis.

## 1. Introduction

Pregnancy represents a unique cardiovascular and pro-thrombotic stress condition characterized by profound physiological adaptations that collectively fulfil Virchow’s triad: hypercoagulability, altered blood flow, and endothelial activation [1]. These changes, primarily aimed at minimizing peripartum hemorrhage, increase the risk of thromboembolic events by four- to five-fold compared with the non-pregnant state and up to 60-fold during the postpartum period, as demonstrated in large population-based studies [2,3]. In high-resource settings, where hemorrhage is effectively managed, thromboembolic disease has consequently emerged as a leading cause of maternal morbidity and mortality, particularly during the third trimester and the postpartum period, when the prothrombotic state reaches its peak [4].

Venous thromboembolism (VTE) is the most frequent manifestation of pregnancy-associated thrombosis, occurring in 0.5–2 per 1000 pregnancies, and has been extensively investigated [3,5,6]. In contrast, arterial thromboembolism remains comparatively underexplored with no standardized diagnostic or therapeutic pathways specifically addressing pregnancy. Although less frequent than VTE, arterial events account for approximately 20% of pregnancy-related vascular thrombotic complications with an incidence of about 4 per 10,000 pregnancies, and contribute disproportionately to severe maternal cardiovascular outcomes [2,7,8].

### 1.1. Pathophysiology of Venous vs. Arterial Thrombosis

Venous and arterial thrombosis are characterized by distinct pathophysiological mechanisms, with substantial differences in the relative contribution of the components of Virchow’s triad, resulting in distinct thrombus composition and important implications for preventive and therapeutic approaches [9].

Venous thrombosis typically develops in the large deep veins of the lower extremities under low-flow conditions. Venous stasis limits the clearance of activated coagulation factors, promoting their local accumulation, sustained thrombin generation, and fibrin formation. Reduced blood flow may also induce local hypoxia and vessel-wall distension, leading to endothelial activation and the expression of adhesion molecules, whereas direct disruption of the vascular wall is generally not required for spontaneous deep-vein thrombosis [10]. Venous thrombogenesis is therefore predominantly coagulation-driven, resulting in thrombi relatively rich in fibrin and erythrocytes, thereby providing the biological rationale for anticoagulation as the cornerstone of VTE prevention and treatment [11].

Conversely, arterial thrombosis develops under high-shear conditions and is more strongly dependent on platelet adhesion, activation, and aggregation. In the general population, the prototypical mechanism is disruption or erosion of an atherosclerotic plaque, resulting in exposure of highly thrombogenic subendothelial components and von Willebrand factor and rapid recruitment of circulating platelets. Activation of the coagulation cascade occurs concurrently, generating thrombin, which both converts fibrinogen into fibrin and acts as a potent platelet agonist, thereby amplifying thrombus formation. Arterial thrombi are therefore generally characterized by a greater platelet contribution, thereby providing the rationale for antiplatelet therapy in the prevention and treatment of many thrombotic arterial events, while the concomitant activation of the coagulation cascade explains the benefit of anticoagulant strategies in selected arterial thrombotic conditions [12,13].

However, thrombus composition may vary in both the arterial and venous circulations according to the underlying etiology, including systemic hypercoagulable states and immunothrombotic mechanisms [14,15,16].

### 1.2. Pathophysiology of Thrombotic Events in Pregnancy

The distinct mechanisms underlying venous and arterial thrombosis are particularly relevant in the context of pregnancy, during which physiological adaptations differentially affect the pathways involved in thrombus formation. The gestational shift toward hypercoagulability, characterized by impaired fibrinolysis, increased levels of fibrinogen and several coagulation factors, enhanced thrombin generation, and reduced endogenous anticoagulant activity, together with venous stasis resulting from compression of the pelvic veins by the enlarging uterus, strongly favors venous thrombogenesis [17].

At the same time, pregnancy induces controlled endothelial activation, immune modulation and low-grade systemic inflammation [18,19,20]. Although these processes are essential for implantation and placental development, they may simultaneously create a vascular environment more susceptible to arterial thrombosis, particularly when additional vascular or systemic risk factors are present [21,22]. In this context, anticoagulant prophylaxis effectively targets the coagulation-dominant mechanisms underlying pregnancy-associated VTE, but cannot be assumed to provide equivalent protection against arterial events [23,24].

The most common arterial events during pregnancy are ischemic stroke and myocardial infarction, often related to arterial dissection [25,26]. Even in otherwise uncomplicated pregnancies, the incidence of these events is significantly increased compared with the non-pregnant state, with a relative risk approximately nine-fold higher for ischemic stroke and three- to four-fold higher for myocardial infarction compared with age-matched non-pregnant women [27,28]. This susceptibility is further amplified in hypertensive disorders of pregnancy, particularly preeclampsia (PE), a syndrome defined by systemic endothelial dysfunction, vascular maladaptation, angiogenic imbalance, placental hypoxia, oxidative stress and further intensified inflammatory and procoagulant activity [21,29,30,31,32,33,34]. Accordingly, PE increases the risk of both ischemic stroke by up to five-fold and myocardial infarction by approximately two-fold compared with normotensive pregnancies [27,35].

Among arterial thrombotic events during pregnancy, acute limb ischemia (ALI), defined as a sudden reduction in arterial perfusion threatening limb viability within 14 days of symptom onset, is exceptionally rare and underreported [36]. We present the first reported case of spontaneous ALI occurring during the first trimester of pregnancy in the absence of structural vascular abnormalities, thrombophilic disorders, or identifiable cardioembolic triggers. Building on this case, we provide a focused narrative review of the available literature on ALI during pregnancy, with particular emphasis on diagnostic and therapeutic challenges, and implications for maternal cardiovascular risk stratification and surveillance within a dedicated cardio-obstetric setting.

## 2. Case Presentation

A 43-year-old woman presented to the Emergency Department with acute-onset left lower limb pain of 48-h duration, rapidly progressing to sensory and motor deficit. At the time of presentation, she was unaware that she was 5 weeks pregnant; the pregnancy was identified during the diagnostic work-up. Her medical history was notable for mild obesity (Body Mass Index 31 kg/m^2^), thalassemia trait, chronic hypertension treated with nebivolol 5 mg/day, type 2 diabetes managed with insulin, and a previous pregnancy complicated by PE. There was no personal or family history of arterial or venous thromboembolism, and she denied preceding claudication, palpitations, or recent trauma. On admission, vital signs were stable: blood pressure 130/82 mmHg, heart rate 87 beats/min, respiratory rate 18 breaths/min, oxygen saturation 100% on room air. Pain intensity was rated 8/10 on the numeric rating scale.

Physical examination revealed a hypothermic left lower limb with absent left pedal and popliteal pulses, while the left femoral pulse was present. Neurological impairment demonstrated sensory loss and mild motor deficit, consistent with Rutherford class IIb ALI. Duplex ultrasonography (DUS) confirmed thrombotic occlusion of the left superficial femoral artery extending to the popliteal segment.

**Figure 1:**
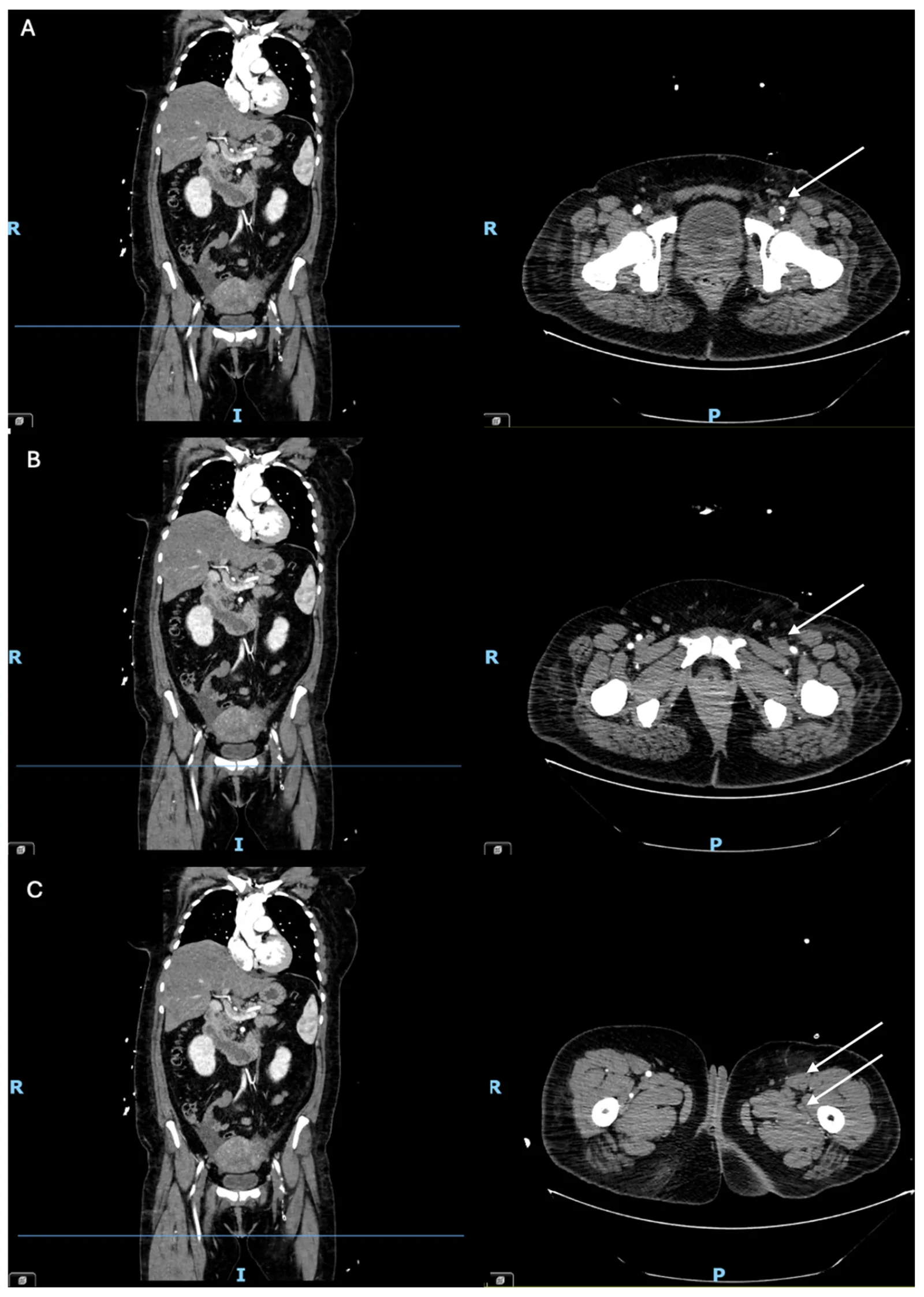
Computed Tomography Angiography performed in the reported case. The arrows indicate intraluminal filling defects in the arteries of the left lower limb on arterial-phase contrast-enhanced Computed Tomography Angiography. In panels (**A**–**C**), the axial image is shown on the right and the corresponding coronal reconstruction on the left; the horizontal blue line in the coronal images indicates the level of the corresponding axial sections. Patient orientation is indicated by R (right), I (inferior), and P (posterior). (**A**) Partial thrombotic occlusion of the left common femoral artery. (**B**) Patency of the proximal left deep femoral artery, with occlusion of the left superficial femoral artery. (**C**) Occlusion of the mid segment of the left deep femoral artery, with persistent occlusion of the left superficial femoral artery.

An intravenous bolus of heparin was promptly administered, followed by urgent surgical thromboembolectomy using a Fogarty catheter. Postoperatively, while continuous intravenous heparin infusion was maintained (1000 IU/h), early re-thrombosis occurred within 24 h. Computed tomography angiography (CTA) (Figure 1) demonstrated extensive femoropopliteal thrombosis without evidence of underlying atherosclerosis or structural vascular abnormalities, necessitating repeat revascularization with a Fogarty catheter.

A bolus of antithrombin III (2000 IU) was administered intraoperatively due to inadequate response to intravenous heparin, resulting in improvement of the Activated Clotting Time from 137 to 200 s. Postoperatively, continuous intravenous heparin was maintained at 1000 IU/h for 72 h, initially requiring additional antithrombin III supplementation to achieve therapeutic anticoagulation (Activated Partial Thromboplastin Time > 2× control). Thereafter, anticoagulation was transitioned to therapeutic-dose low-molecular-weight heparin (LMWH; 10,000 IU subcutaneously twice daily), and low-dose aspirin (100 mg daily) was initiated. Due to postoperative anemia and inadequate spontaneous hemoglobin recovery over the following weeks, further compounded by the patient’s underlying thalassemia trait, supportive treatment with intravenous iron supplementation and blood transfusion was administered, resulting in subsequent improvement in hemoglobin levels.

A comprehensive etiological workup, including transthoracic echocardiography, transcranial Doppler ultrasound, Holter electrocardiography and thrombophilia screening, was performed and did not identify any cardioembolic source or inherited prothrombotic condition (Table 1). Borderline anticardiolipin IgG positivity (IgG 17 U/mL, IgM 3 U/mL) was detected but did not meet diagnostic criteria for antiphospholipid syndrome.

A trimesterly follow-up was performed at the Cardio-obstetric Clinic of Our Institution for close monitoring of maternal hemodynamics. Serial echocardiographic assessments performed during pregnancy revealed hypertensive concentric hypertrophy and a trend towards a hyperdynamic circulatory state (at 30 weeks of gestation systemic vascular resistance was 761.9 dyn·s·cm^−5^ and cardiac output was 9.1 L/min). Laboratory and anthropometric parameters were also assessed serially, with values summarized in Table 2.

At 35 weeks of gestation, the patient developed superimposed PE, diagnosed according to the 2021 International Society for the Study of Hypertension in Pregnancy (ISSHP) criteria, based on worsening blood pressure control, progressive proteinuria and evidence of uteroplacental dysfunction. Delivery was performed at 37 weeks by elective cesarean section. The postpartum course was complicated by severe hemorrhage requiring transfusion, despite therapeutic LMWH having been discontinued 24 h before surgery. Enoxaparin was restarted at full therapeutic dosing on postoperative day 3 according to clinical stability, which was maintained throughout follow-up. Surveillance was initially conducted on a monthly basis with repeat DUS, and subsequently every six months, with no evidence of recurrent thrombotic events. Anticoagulation was discontinued four months postpartum after confirmation of sustained arterial recanalization and a negative thrombophilia screening.

Overall, the patient presented with multiple clinical cardiovascular risk factors, including advanced maternal age, a history of PE, hypertension, obesity, and type 2 diabetes. These conditions may have contributed to endothelial dysfunction and vascular vulnerability within the prothrombotic milieu of pregnancy.

## 3. Narrative Review

### 3.1. Epidemiology

The incidence of ALI in the general population is estimated at approximately 4–12 cases per 100,000 person-years and is substantially higher among patients with peripheral arterial disease (PAD), in whom annual rates range from 0.8% to 1.7% [37,38].

However, accurate incidence data during pregnancy are lacking. In a large cohort analyzed by DeCarlo et al. uncomplicated pregnancy was associated with an increased risk of acute peripheral arterial events with a hazard ratio (HR) of 1.85, especially within the first four weeks postpartum, while the presence of hypertensive disorders of pregnancy further increased this risk, with mild-to-moderate PE (HR 8.31) and severe PE (HR 14) emerging as strong independent predictors. Among approximately 280,000 included pregnancies, only 24 acute arterial events were recorded, of which only one was ALI due to an external iliac artery occlusion occurring within four weeks postpartum [39].

Consistent with the rarity of ALI in the obstetric setting, most of the available evidence derives from isolated case reports or small case series [40]. Nevertheless, evidence from the general PAD population underscores its potentially severe prognosis and the systemic vascular burden associated with ALI. Even after timely revascularization, patients with ALI exhibited a 41.9% cumulative risk of major amputation or repeat peripheral revascularization and a 9.8% risk of myocardial infarction or stroke over a median follow-up of 2.7 years. Limb-related events were most frequent within the first year following the index procedure, whereas cardiovascular risk persisted and continued to accrue over time [41,42].

### 3.2. Diagnostic Approach of Acute Limb Ischemia in Pregnancy

The clinical presentation of ALI is characterized by the “six P’s”: pain, pulselessness, pallor, poikilothermia, paresthesia, and paralysis. Symptoms typically evolve rapidly, with early sensory deficits, such as impaired fine touch and proprioception, progressing to anesthesia and subsequent motor dysfunction within hours as a consequence of advancing ischemic injury to muscle and nerve tissue [43].

A focused vascular and neurological examination is essential both to establish clinical suspicion and to stratify severity according to the Rutherford classification [44]. However, diagnosis may be challenging in selected cases, as symptoms can mimic traumatic or neurological conditions, including Guillain–Barré syndrome or transverse myelitis [40,45].

Given the limited accuracy of pulse palpation alone, a handheld continuous-wave Doppler device is often used to rapidly confirm the diagnosis and refine severity assessment, as the absence of an arterial Doppler signal suggests a threatened limb, whereas the absence of both arterial and venous signals suggests advanced ischemia with limited salvage potential [46].

Prompt recognition is critical to prevent irreversible limb damage and optimize maternal and fetal outcomes and, although non-invasive vascular imaging (DUS, CTA) may assist in diagnostic confirmation or procedural planning, one must not delay urgent revascularization [47,48,49,50].

In pregnancy, imaging decisions require careful consideration of fetal safety. While minimizing radiation exposure during pregnancy is preferred to reduce potential teratogenic, carcinogenic, or mutagenic effects, particularly during organogenesis, current recommendations emphasize that necessary imaging should not be withheld in life- or limb-threatening conditions when results are expected to influence management [51].

Fetal radiation exposure from CTA varies according to the anatomical region examined; however, most diagnostic CT procedures are associated with doses well below 50 mGy, the threshold above which deterministic effects such as congenital malformations, pregnancy loss, or neurodevelopmental impairment may occur, while iodinated contrast crosses the placenta but has not been associated with teratogenic effects [52,53,54].

Whenever feasible, low-dose protocols should be implemented in accordance with the ALARA (“as low as reasonably achievable”) principle to further minimize fetal exposure. However, stochastic risks, including a small increase in childhood cancer, particularly leukemia, are dose-dependent and may persist even at low radiation exposures, despite being very low at typical diagnostic doses [55,56,57].

### 3.3. Etiology of Acute Limb Ischemia in Pregnancy

**Figure 2:**
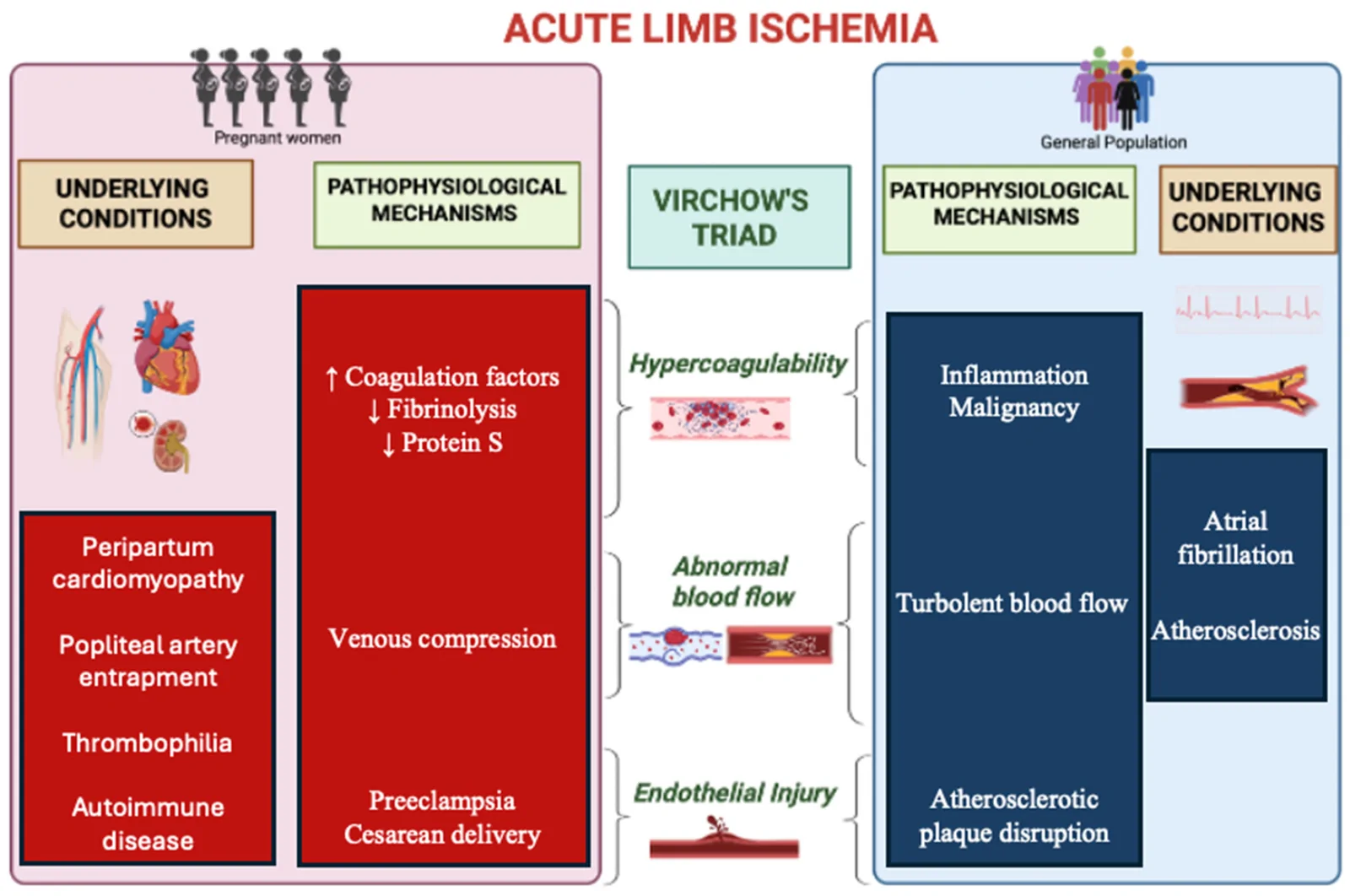
Pathophysiological mechanisms and underlying conditions associated with acute limb ischemia in pregnancy and in the general population. As shown in the central panel, vascular thrombosis is classically explained by Virchow’s triad. Pregnancy ((**Left**) panel, red) is characterized by increased coagulation factors, venous stasis related to uterine compression of the iliac veins, and endothelial dysfunction, which may be further enhanced by preeclampsia or cesarean delivery. In contrast, in the general population ((**Right**) panel, blue), arterial thrombosis is predominantly associated with endothelial injury following atherosclerotic plaque disruption. Embolic acute limb ischemia is most commonly of cardiac origin, primarily related to atrial fibrillation in the general population and to peripartum cardiomyopathy during pregnancy.

ALI results from sudden arterial occlusion caused by either thrombotic or embolic events. The etiology of ALI in pregnancy differs markedly from that observed in the general population, where atrial fibrillation and atherosclerosis represent the leading causes (Figure 2) [44].

In young pregnant women, the arterial tree is generally free from significant atherosclerotic disease or chronic structural pathology [58]. Accordingly, despite the intrinsic prothrombotic state of pregnancy, embolic mechanisms represent the most frequently reported cause of ALI in this population [59].

Most cases occur in the postpartum period, with peripartum cardiomyopathy (PPCM) representing the most frequent underlying cause and thromboembolic events reported in approximately 5–9% of patients with PPCM. In this condition, left ventricular systolic dysfunction promotes intracavitary thrombus formation, detected in 10–17% of echocardiograms, and acts synergistically with the hypercoagulable state of pregnancy and the puerperium [60]. Notably, PPCM frequently clusters with PE, an additional independent risk factor for thrombotic events [61].

Another relevant cause of pregnancy-associated cardioembolic ALI is iatrogenic arterial injury following obstetric procedures, such as arterial embolization and prophylactic balloon catheterization in the management of obstetric hemorrhage [62].

In contrast, cases of ALI due to in-situ arterial thrombosis in pregnant women are exceedingly rare. When reported, they have occurred intrapartum or postpartum and have almost invariably been associated with at least one predisposing condition, such as nephrotic syndrome or autoimmune vasculitis [63,64].

Conversely, early-gestation arterial thrombosis in the absence of predisposing conditions remains virtually undocumented, and the only case of thrombotic ALI described during the first trimester was secondary to popliteal artery entrapment syndrome [65].

Aortic dissection, although rare, should also be considered in the differential diagnosis of pregnancy-associated ALI. It represents a relevant cause of severe arterial complications during gestation, accounting for approximately 20% of arterial dissections, after carotid and vertebral involvement, and occurs with a 4–25-fold higher incidence compared with age-matched non-pregnant women, particularly in the presence of multifetal gestation or PE. Most events arise in the third trimester or early postpartum period, when peak hemodynamic stress and pregnancy-related hormonal changes promote structural alterations of the vascular wall [66,67,68]. Although classically presenting with acute chest or back pain, aortic dissection may also manifest with peripheral malperfusion syndromes, underscoring the need for a broad differential diagnosis in pregnant or postpartum patients presenting with acute limb symptoms [69].

Paradoxical embolism is a recognized cause of cryptogenic arterial embolism presenting as ALI in the presence of intracardiac or intrapulmonary right-to-left shunts. A systematic review identified 51 reported cases of patent foramen ovale (PFO)-mediated ALI in the non-pregnant population, predominantly affecting the lower limbs and left-sided arterial circulation [70]. Although no cases of ALI secondary to paradoxical embolism have been reported during pregnancy, case reports describe other arterial events, such as myocardial infarction and ischemic stroke, attributed to PFO in pregnant women, even during the first trimester [71,72,73].

To date, no cases of maternal ALI during pregnancy clearly attributable to inherited thrombophilic mutations have been reported despite such association being described in the general population, since its link with major arterial events is modest and largely outweighed by traditional cardiovascular risk factors [74].

### 3.4. Treatment of Acute Limb Ischemia

According to the 2020 European Society for Vascular Surgery (ESVS) and the 2024 American College of Cardiology (ACC) guidelines, the cornerstone of ALI management consists of immediate systemic anticoagulation with intravenous unfractionated heparin to prevent thrombus propagation, followed by urgent revascularization in patients with a salvageable limb, whereas primary amputation is indicated in cases of Rutherford III ischemia [44,75,76].

The choice between surgical and endovascular revascularization should be individualized according to clinical severity, anatomical considerations, and local expertise. Although catheter-directed thrombolysis was initially used primarily in patients with non-immediately threatened limbs (Rutherford I–IIa) and surgical thromboembolectomy using a Fogarty catheter preferred in cases of severe ischemia, meta-analyses and retrospective studies have demonstrated no significant differences in mortality or limb salvage between the two approaches. Accordingly, current guidelines consider both strategies appropriate options for the treatment of salvageable limbs, despite a higher bleeding risk associated with thrombolysis [44,75,76]. Advanced percutaneous techniques, including ultrasound-assisted thrombolysis, mechanical thrombolysis, thrombo-aspiration are being progressively refined, with limb salvage rates exceeding 80% [75,77].

Pregnant and breastfeeding women have historically been excluded from clinical trials evaluating therapeutic strategies, resulting in reliance on registries and expert consensus. Neither the 2020 ESVS nor the 2024 ACC guidelines provide specific recommendations for the management of arterial ALI in pregnancy. However, a position statement from the Society of Interventional Radiology on acute deep vein thrombosis acknowledges the technical feasibility of catheter-directed thrombolysis during pregnancy, while also highlighting non-negligible risks, including major maternal bleeding, preterm delivery, maternal death, and fetal complications, including fetal loss, underscoring the need for careful patient selection [78]. In this context, endovascular thrombolysis may be considered in severe limb-threatening presentations, particularly when anticoagulation alone is insufficient [79]. When performed, radiation exposure should be minimized through low-dose protocols and abdominal lead shielding, and first-trimester procedures should be avoided whenever possible because of increased teratogenic vulnerability [78,79].

Although these data derive largely from venous thromboembolism rather than arterial ALI, they support catheter-directed thrombolysis as a potential therapeutic option in carefully selected cases.

However, in the cases of arterial ALI reported in the literature, open surgical thromboembolectomy remains the preferred revascularization strategy, with high limb salvage rates and favorable maternal outcomes [59]. Less severe or more limited presentations have, in selected cases, been successfully managed with anticoagulation alone, with documented spontaneous recanalization [80].

In the absence of randomized evidence and dedicated arterial ALI guidelines for pregnancy, therapeutic decisions must rely on individualized multidisciplinary evaluation, with preference for anticoagulation and open surgical revascularization in most patients, although pregnancy itself does not represent an absolute contraindication to endovascular procedures.

### 3.5. Diagnostic Workup After Revascularization

The post-acute diagnostic workup after ALI is essential to identify the underlying etiology and mitigate the risk of recurrent cardiovascular events, and should include a systematic assessment for cardioembolic sources, arterial structural abnormalities, and prothrombotic conditions [75].

Because PAD represents one of the most common causes of ALI in the general population, a targeted history should explore baseline limb symptoms and prior lower-extremity revascularization procedures. Other major mechanisms include native vessel thrombosis and systemic embolization; therefore, evaluation must investigate atrial fibrillation, recent myocardial infarction, cardiomyopathy, paradoxical embolism, infective endocarditis, valvular heart disease, aortic pathology and a recent history of arterial access procedures [44].

Transthoracic echocardiography and electrocardiographic monitoring are recommended to exclude intracardiac thrombus or arrhythmias, with extended rhythm monitoring considered in selected cases, while vascular imaging should evaluate for dissection, aneurysmal disease, or underlying PAD and may also assess residual stenosis following revascularization [44,81]. Although transthoracic echocardiography can detect structural septal defects, PFO diagnosis may be challenging and typically requires a bubble study with agitated saline injection to demonstrate a right-to-left intracardiac shunt, while Transcranial Doppler offers a complementary assessment for cryptogenic arterial embolism, with the advantage of detecting right-to-left shunts irrespective of their anatomical location. However, there are conflicting opinions regarding the use of agitated saline contrast in pregnant patients, owing to both the lack of systematic safety data and the theoretical risk of embolic complications [82].

In the absence of an identifiable cause, in-situ thrombosis related to systemic prothrombotic conditions should be considered, including vasculitis, active malignancy, or drug-induced states [44,83].

Among acquired thrombophilia, antiphospholipid syndrome (APS) is consistently associated with both venous and arterial thrombosis, particularly ischemic stroke (HR 1.76) and myocardial infarction, often occurring in younger patients and in the absence of obstructive coronary artery disease [84,85]. Although ALI has not been systematically assessed in the context of APS, a meta-analysis by Merashli et al. demonstrated that antiphospholipid antibodies are significantly associated with lower-extremity PAD, critical limb ischemia, and revascularization failure [86]. Similarly, antiphospholipid antibodies have been detected in approximately 13.5% of pregnant women with ischemic stroke and in 11% of those with pregnancy-associated myocardial infarction, underscoring their potential contribution to arterial thrombotic risk during gestation and supporting appropriate evaluation of APS in patients presenting with unexplained arterial thrombosis [87,88,89,90]. However, transient antiphospholipid antibody positivity has also been described in otherwise uncomplicated pregnancies, and its clinical significance in the absence of persistent elevation remains uncertain [91].

Despite being strongly associated with VTE, the contribution of inherited thrombophilia to arterial thrombosis is uncertain, and available evidence derives primarily from studies on ischemic stroke and myocardial infarction, while ALI has not been evaluated as an independent endpoint [90]. In the general population, only modest associations with ischemic stroke have been reported for factor V Leiden with an Odds Ratio (OR) of 1.25, prothrombin G20210A mutation (OR 1.48), protein C deficiency (OR 2.13), and protein S deficiency (OR 2.26), whereas no significant association was found for antithrombin deficiency [92].

Data in pregnancy are even more limited. While the impact of inherited thrombophilia on the risk of a first venous thromboembolic event during pregnancy is well established, particularly in women with antithrombin, protein C, or protein S deficiency, or homozygous factor V Leiden, its role in arterial thrombosis during gestation remains insufficiently explored [93]. A case–control study reported inherited thrombophilia in 83% of women with transient ischemic neurological events during pregnancy compared with 17% of controls, supporting a potential association between thrombophilia and pregnancy-related ischemic events [94,95].

However, routine thrombophilia screening is not recommended in unselected patient populations with myocardial infarction, ischemic stroke or ALI, since population-based screening of pregnant women has not been shown to be cost-effective [96]. Testing may be considered in selected cases, particularly when the arterial event is unprovoked, occurs at a young age in the absence of conventional risk factors, or when there is a strong family history of thrombosis [97,98,99].

Moreover, interpretation of thrombophilia screening during pregnancy requires caution, as physiological gestational adaptations alter reference ranges for coagulation factors and anticoagulant proteins. Protein S levels decline physiologically, while protein C values may fluctuate, making it difficult to distinguish true deficiency from normal pregnancy-related changes [100,101]. Acute thrombosis may further affect functional assays, leading to false-positive or false-negative results [102].

Accordingly, current guidelines recommend deferring thrombophilia testing until at least six weeks postpartum and off anticoagulation to avoid misclassification and inappropriate management [101].

### 3.6. Follow-Up After Revascularization

Patients with ALI are at high risk of recurrent major cardiovascular events, frequently resulting in rehospitalization, repeat revascularization, and increased early mortality; therefore, appropriate post-procedural follow-up is essential to improve clinical outcomes [103]. Post-procedural management includes close monitoring for compartment syndrome and structured vascular surveillance. Follow-up relies primarily on DUS, the non-invasive gold standard for detecting restenosis or recurrent thrombosis, whereas CTA is not recommended routinely and should be reserved for selected cases with new-onset vascular symptoms, without delaying urgent revascularization when required [104].

After revascularization, intravenous anticoagulation should be continued and subsequently transitioned to long-term antithrombotic therapy tailored to the underlying etiology for secondary prevention [103]. However, robust evidence supporting the optimal duration of anticoagulation after ALI is limited.

While long-term anticoagulation clearly reduces embolic risk in patients with atrial fibrillation and mitigates thrombotic risk in thrombophilia following an ALI event, its role in patients without an identifiable cause remains uncertain, particularly regarding prevention of recurrent ALI and limb-related outcomes; moreover, the potential added benefit of combining anticoagulation with antiplatelet therapy warrants further evaluation in this specific patient population, which often lacks underlying PAD [75,105,106].

In the absence of pregnancy-specific recommendation for ALI, management is individualized, taking into account the available evidence from the thromboembolic literature, which supports continuation of anticoagulation throughout pregnancy and for at least six weeks postpartum, ensuring a minimum total duration of three months after a VTE event [107,108,109]. According to the 2023 American Society of Hematology guidelines, patients with pregnancy- or puerperium-provoked VTE should undergo thrombophilia testing and receive indefinite anticoagulant therapy if high-risk thrombophilia is confirmed [110].

In pregnancy, anticoagulation strategies are constrained by safety considerations. Because of the teratogenicity of warfarin and the lack of safety data for direct oral anticoagulants (DOACs), during gestation therapeutic-dose LMWH calculated on early pregnancy body weight remains the treatment of choice, aiming for 4–6 h peak anti-Xa values of 0.6–1.2 IU/mL, while DOACs should also be avoided during breastfeeding [101,107,111].

Pregnancy also profoundly modifies both the pharmacokinetics and pharmacodynamics of heparin. Expansion of plasma volume and increased renal clearance may reduce circulating drug concentrations [112]. Moreover, the physiological reduction in antithrombin during uncomplicated pregnancy, and its further decline in pregnancies complicated by PE, may attenuate the anticoagulant effect of heparin, which acts by potentiating endogenous antithrombin activity, thereby contributing to heparin resistance in selected cases [113,114,115]. Consequently, heparin therapy during pregnancy often requires dose adjustments and closer laboratory monitoring, and in cases of therapeutic resistance, supplementation with antithrombin concentrate may restore heparin responsiveness [116,117].

Despite the established association between thrombotic events and other cardiometabolic complications of pregnancy, such as PE or PPCM, there are currently no specific recommendations for close pregnancy follow-up after arterial thrombosis, and monitoring strategies are often left to the discretion of the treating physician [59,118,119].

In this context, a structured multidisciplinary cardio-obstetric approach may help standardize care and improve maternal and fetal outcomes in this high-risk population, as suggested by observational studies reporting fewer maternal complications compared with standard care in pregnancies at high cardiovascular risk, through comprehensive risk stratification, optimization of medical therapy, coordinated delivery planning, and structured postpartum surveillance [120,121,122,123].

In high-risk pregnancies, including those complicated by prior arterial thrombosis, serial echocardiographic assessment enables monitoring of maternal hemodynamic adaptation and early identification of maladaptive phenotypes that may precede the clinical onset of PE, allowing timely initiation or intensification of antihypertensive therapy and potentially reducing the risk of hypertensive crises, maternal heart failure, ischemic events, and renal dysfunction, while improving neonatal outcomes [27,124]. Echocardiography also enables early recognition of PPCM and intracardiac thrombus, thereby reducing embolic risk [60].

In pregnant women receiving therapeutic anticoagulation, careful peripartum management of treatment interruption and resumption is essential to balance thrombotic and hemorrhagic risks. Although therapeutic-dose LMWH is associated with an increased risk of postpartum hemorrhage after vaginal delivery compared with non-anticoagulated women, this risk does not appear to be significantly increased in the setting of planned cesarean delivery, particularly when LMWH is discontinued at least 24 h before scheduled delivery, as recommended by current guidelines [108,125,126]. Anticoagulation should generally be resumed 6–12 h after vaginal delivery and 12–24 h after cesarean section, since premature reinitiation is associated with a higher risk of postpartum bleeding and wound-related complications, including surgical site hematoma [125,127].

### 3.7. Primary Prevention of Thrombotic Events in Pregnancy

During pregnancy and the puerperium, women are at increased risk of thrombosis, and multiple clinical and anamnestic factors may further modulate this risk. Beyond prothrombotic conditions recognized in the general population, such as inherited thrombophilia, personal or family history of VTE, obesity, nephrotic syndrome, autoimmune or rheumatologic diseases, diabetes, hypertension, immobilization, and smoking, pregnancy-specific factors including advanced maternal age, multiple gestation, parity ≥3, gestational diabetes, PE, hyperemesis, post-partum haemorrhage, assisted reproductive techniques, cesarean delivery, and infection should be systematically integrated into a risk-stratified approach to guide thromboprophylaxis [4,128,129]. However, high-quality evidence to inform pharmacologic thromboprophylaxis during pregnancy remains limited and is largely derived from observational data, as adequately powered randomized trials in pregnant populations are lacking [130,131]. Current recommendations primarily address VTE, with the strongest evidence supporting prophylactic LMWH in high-risk women throughout pregnancy and for six weeks postpartum, particularly in those with prior unprovoked or estrogen-provoked VTE or those with high-risk thrombophilia with a history of prior VTE [132,133,134]. Moreover, adherence to these recommendations remains suboptimal, even in the United Kingdom, where it has been reported that over 80% of women who died from VTE had identifiable thromboembolic risk factors that were either not recognized or inadequately managed [128].

The continuous increase in the prevalence of clinical thrombotic risk factors over recent decades within the obstetric population, further underscores the need for a comprehensive risk-based strategy to improve maternal outcomes [135,136]. This risk–benefit balance is even more delicate in the antepartum period, where unpredictable obstetric events, such as spontaneous labor, neuraxial anesthesia placement, or emergent cesarean delivery, require timely interruption of anticoagulation, and inadequate suspension of LMWH may adversely affect maternal outcomes [130].

Besides thrombosis prevention, the use of LMWH may also reduce the incidence of PE in selected high-risk women, particularly when combined with low-dose aspirin and initiated early in pregnancy, although current guidelines do not recommend its routine use for this indication [137,138,139].

**Figure 3:**
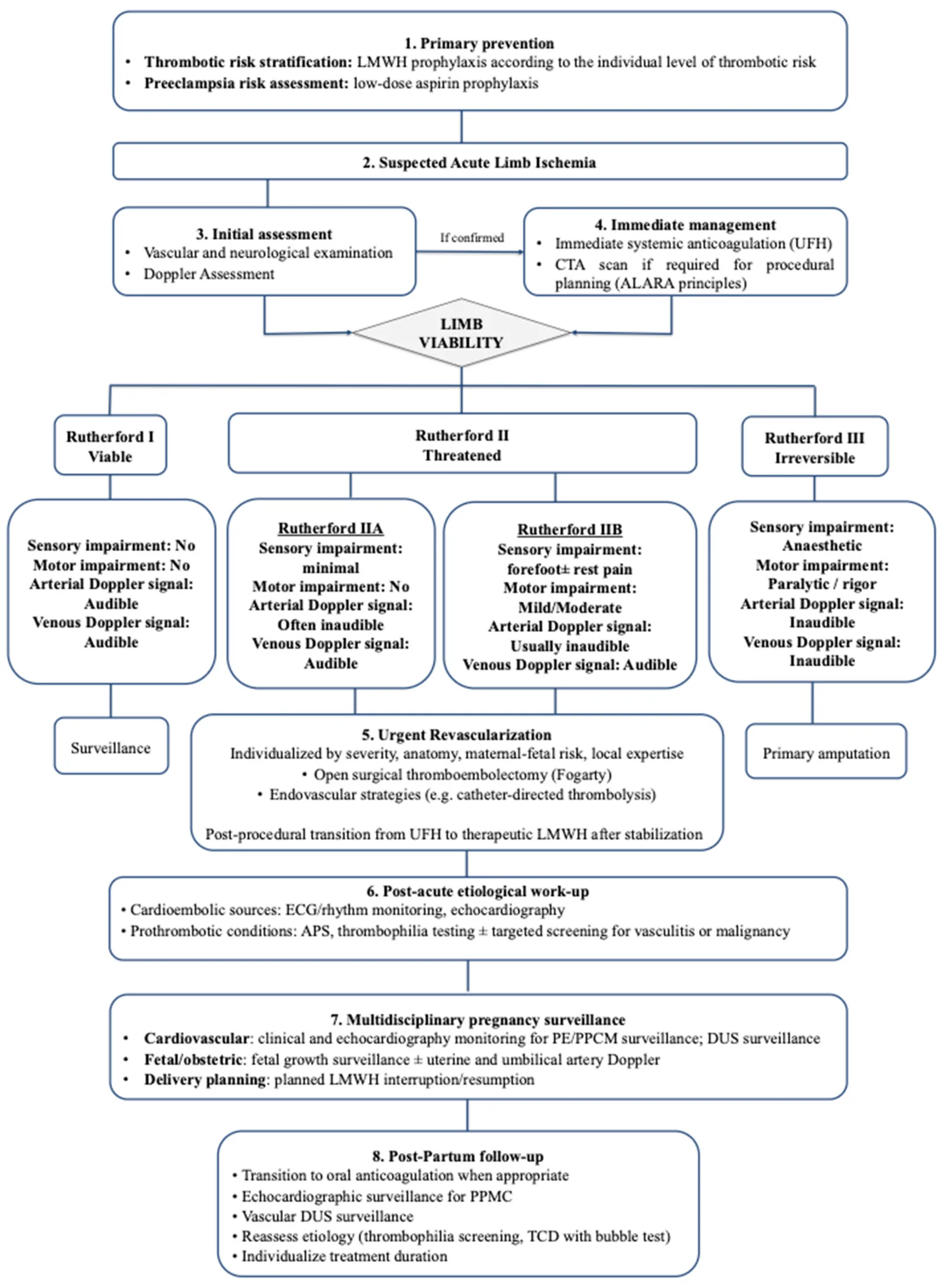
Proposed multidisciplinary management framework for acute limb ischemia during pregnancy. The proposed flowchart summarizes a multidisciplinary approach to the prevention, diagnosis, acute management, and longitudinal follow-up of acute limb ischemia during pregnancy. Given the absence of dedicated guidelines for acute limb ischemia during pregnancy, the proposed framework integrates recommendations for acute limb ischemia in the general population with pregnancy-specific considerations regarding antithrombotic therapy, maternal and fetal surveillance, delivery planning, and postpartum follow-up. Abbreviations: ALARA, as low as reasonably achievable; ALI, acute limb ischemia; APS, antiphospholipid syndrome; BP, blood pressure; CTA, computed tomography angiography; DUS, duplex ultrasound; ECG, electrocardiography; LMWH, low-molecular-weight heparin; PAD, peripheral artery disease; PE, preeclampsia; PPCM, peripartum cardiomyopathy; TCD, transcranial doppler; UFH, unfractionated heparin.

Figure 3 summarizes the proposed multidisciplinary management framework for acute limb ischemia during pregnancy.

## 4. Conclusions

ALI during pregnancy is rare but clinically critical. Although most evidence derives from case reports, pregnancy-related hypercoagulability and hemodynamic changes, particularly in the presence of hypertensive disorders, may lower the threshold for arterial thrombosis.

The absence of dedicated guidelines for arterial ALI in pregnancy reflects a significant gap of knowledge. Management is largely extrapolated from non-pregnant populations and VTE data, necessitating individualized, multidisciplinary decision-making. Early diagnosis, prompt revascularization, and carefully balanced anticoagulation remain central to optimizing maternal and limb outcomes.

This case demonstrates that arterial events may occur as early as the first trimester, even in the absence of structural vascular abnormalities, thrombophilic disorders, or cardioembolic triggers, while clinical and pregnancy-specific conditions may synergistically amplify thrombotic susceptibility within the prothrombotic milieu of gestation. We therefore highlight the importance of both comprehensive thrombotic risk stratification and a high index of suspicion in pregnant women presenting with ALI symptoms to prevent delay and reduce morbidity.

## Figures and Tables

**Table 1:** Diagnostic work-up for potential causes of arterial thrombosis and cardioembolism.

Diagnostic Work-Up				
Thrombophilic screening			**Results**	**Normal Range**
		Lupus anticoagulants: Diluted phospholipids (APTT)	1.14	<1.25 negative
		Lupus anticoagulants: Diluted phospholipids (DRVVT)	1.04	<1.25 negative
		Anti-beta-2 glycoprotein I	IgG 2 U/mL IgM 4 U/mL	<7 U/mL negative 7–10 U/mL borderline >10 U/mL positive
		Anticardiolipin	IgG 17 U/mL IgM 3 U/mL	<10 U/mL negative 10–40 U/mL borderline >40 U/mL positive
		Antithrombin III activity	106%	>80%
		Fibrinogen	309 mg/dL	200–400 mg/dL
		Hepatoquick	1.12	<1.2
		Factor II	1.10 U/mL	>0.7 U/mL
		Factor V activity	155.9%	>70%
		Factor VII	0.93 U/mL	>0.7 U/mL
		Factor VIII	4.529 U/mL	>0.7 U/mL
		Von Willebrand factor	2.51 U/mL	>0.7 U/mL
		Factor IX	1.664 U/mL	>0.7 U/mL
		Factor X	1.17 U/mL	>0.7 U/mL
		Factor XI activity	131%	>70%
		Factor XII activity	67%	>70%
		Protein C activity	105%	70–140%
		Protein S activity	73%	60–140%
Instrumental investigations	Thoraco-abdominal CTA	No diffuse atheromatosis. No arterial collateral branches		
	Holter ECG	Sinus rhythm with occasional supraventricular ectopic beats		
	TCD with bubble test	Negative for right-to-left shunt (no transient high-intensity signals at baseline or after the Valsalva maneuver).		
	Echocardiogram	No intracardiac thrombus. No significant valvular dysfunction. Concentric left ventricular hypertrophy and mild-to-moderate diastolic dysfunction.		

Coagulation factor testing reported in this table was performed two weeks after the acute thrombotic event. Antithrombin III activity was reassessed one month after the acute event to minimize the potential influence of antithrombin III supplementation administered during the acute phase and was confirmed to be within the normal range (Antithrombin III activity 100%). The fibrinogen value reported in this table was measured more than three months after the acute thrombotic event. Abbreviations: CTA, Computed Tomography Angiography; ECG, Electrocardiography; TCD, Transcranial Doppler; APTT, Activated Partial Thromboplastin Time; DRVVT, Dilute Russell’s Viper Venom Time.

**Table 2:** Longitudinal clinical and laboratory parameters in the reported case.

		Unit of Measurement	Normal Range	Before Pregnancy	11 Weeks of Gestation	28 Weeks of Gestation	34 Weeks of Gestation
**Anthropometric and hemodynamic parameters**	**Weight**	kg	-	93	96	103.5	108
	**Height**	cm	-	175	-	-	-
	**BMI**	kg/m^2^	18.5–24.9	30.37	31.35	33.80	35.27
	**SBP**	mmHg	Optimal < 120	110	120	130	120
	**DBP**	mmHg	Optimal < 80	70	70	80	75
**Hematological parameters**	**Leukocytes**	10^9^/L	3.6–10.5	n.r.	8.24	n.r.	6.76
	**Erythrocytes**	10^12^/L	3.9–5.2	n.r.	3.39	n.r.	4.71
	**Hemoglobin**	g/dL	12.0–15.6	n.r.	8.7	n.r.	11.6
	**Hematocrit**	%	35.5–45.5	n.r.	26.2	n.r.	36.1
	**MCV**	fL	80–99	n.r.	77	n.r.	77
	**MCH**	pg	27.0–33.5	n.r.	25.7	n.r.	24.6
	**MCHC**	g/dL	31.5–36.0	n.r.	33.2	n.r.	32.1
	**RDW**	%	11.5–15.0	n.r.	18.8	n.r.	21.9
	**Platelets**	10^9^/L	160–370	n.r.	233	n.r.	322
	**MPV**	fL	8.5–11.5	n.r.	9.3	n.r.	9.7
**Renal function**	**Creatinine**	mg/dL	0.5–1.2	n.r.	0.39	n.r.	0.43
	**eGFR (CKD-EPI)**	mL/min/1.73 m^2^	-	n.r.	129.96	n.r.	124.97
	**Urea**	mg/dL	17–43	n.r.	17	n.r.	19
	**Uric acid**	mg/dL	2.4–5.7	n.r.	4.6	n.r.	4.1
	**Sodium**	mmol/L	136–145	n.r.	139	n.r.	134
	**Potassium**	mmol/L	3.5–5.3	n.r.	4.3	n.r.	4.2
	**Calcium**	mg/dL	8.6–10.5	n.r.	9.1	n.r.	8.3
**Liver function**	**Total Serum Protein**	g/L	66–83	n.r.	70	n.r.	49
	**Serum Albumin**	g/L	35–50	n.r.	n.r.	n.r.	28.5
	**AST**	UI/L	<35	n.r.	12	n.r.	12
	**ALT**	UI/L	<35	n.r.	6	n.r.	8
	**GGT**	UI/L	<38	n.r.	18	n.r.	14
	**FIB-4 score**	-	Low risk < 1.3	0.35	0.33	0.55	0.48
	**NAFLD Fibrosis Score**	-	Low risk < −1.455	−0.99	n.r.	−3.82	−1.05
**Lipid profile**	**Total cholesterol**	mg/dL	Low risk < 200	220	208	190	n.r.
	**HDL**	mg/dL	>43	48	63	61	n.r.
	**LDL**	mg/dL	Optimal < 100	124	113.4	94.8	n.r.
	**Non-HDL cholesterol**	mg/dL	Optimal < 130	172	145	129	129
	**Triglycerides**	mg/dL	<200	240	158	171	n.r.
**Glucose metabolism**	**Fasting blood glucose**	mg/dL	60–110	198	215	148	96
	**Glycated Hemoglobin**	mmol/mol	20–42	80	65	73	n.r.
	**HbA1c**	%	<5.7	9.47	8.1	8.83	n.r.
	**Fructosamine**	micromol/L	200–285	306	n.r.	n.r.	n.r.
**Urinary parameters**	**Albuminuria**	mg/dL	<30	95	102	159	150
	**Ketone bodies**	mg/dL	negative	0	n.r.	5	n.r.
	**Glycosuria**	mg/dL	negative	250	n.r.	500	n.r.
	**Total urinary proteins**	mg/24 h	<150	250	375	552	640
**Thyroid function**	**TSH**	mUI/L	0.4–4	n.r.	1.01	1.33	n.r.
	**FT4**	pg/mL	10–22	n.r.	11.5	12.7	n.r.
	**FT3**	pg/mL	2.3–4.2	n.r.	2.6	n.r.	n.r.
**Iron status and hematinic parameters**	**Folic acid**	ng/mL	4–20	n.r.	5	4.1	n.r.
	**Iron**	microgr/dL	50–170	n.r.	20	21	63
	**Transferrin**	mg/dL	200–360	n.r.	375	424	333
**Other biochemical parameters**	**ESR**	mm/h	<20	n.r.	22	n.r.	n.r.
	**CPK**	UI/L	<170	n.r.	35	n.r.	35

Clinical and laboratory parameters across different gestational ages are reported. Abbreviations: ALT, Alanine Aminotransferase; AST, Aspartate Aminotransferase; BMI, Body Mass Index; CKD-EPI, Chronic Kidney Disease Epidemiology Collaboration; CPK, Creatine Phosphokinase; DBP, Diastolic Blood Pressure; eGFR, Estimated Glomerular Filtration Rate; ESR, Erythrocyte Sedimentation Rate; FIB-4, Fibrosis-4 Index; FT3, Free Triiodothyronine; FT4, Free Thyroxine; GGT, Gamma-Glutamyl Transferase; HbA1c, Glycated Hemoglobin; HDL, High-Density Lipoprotein; LDL, Low-Density Lipoprotein; MCH, Mean Corpuscular Hemoglobin; MCHC, Mean Corpuscular Hemoglobin Concentration; MCV, Mean Corpuscular Volume; MPV, Mean Platelet Volume; NAFLD, Non-Alcoholic Fatty Liver Disease; n.r., not recorded; RDW, Red Cell Distribution Width; SBP, Systolic Blood Pressure; TSH, Thyroid-Stimulating Hormone.

## Data Availability

The data supporting the findings of this study are included within the article. Further data are not publicly available due to patient privacy and confidentiality.

## Abbreviations

ALI	Acute Limb Ischemia
APS	Antiphospholipid Syndrome
ATE	Arterial Thromboembolism
BMI	Body Mass Index
CTA	Computed Tomography Angiography
DOAC	Direct Oral Anticoagulant
DUS	Duplex Ultrasound
ECG	Electrocardiogram
ESC	European Society of Cardiology
HR	Hazard Ratio
LMWH	Low-Molecular-Weight Heparin
OR	Odds Ratio
PAD	Peripheral Arterial Disease
PE	Preeclampsia
PFO	Patent Foramen Ovale
PPCM	Peripartum Cardiomyopathy
RCOG	Royal College of Obstetricians and Gynaecologists
TCD	Transcranial Doppler
VTE	Venous Thromboembolism
